# The United States’ contribution of plastic waste to land and ocean

**DOI:** 10.1126/sciadv.abd0288

**Published:** 2020-10-30

**Authors:** Kara Lavender Law, Natalie Starr, Theodore R. Siegler, Jenna R. Jambeck, Nicholas J. Mallos, George H. Leonard

**Affiliations:** 1 Sea Education Association, Woods Hole, MA, USA.; 2 DSM Environmental Services, Windsor, VT, USA.; 3 College of Engineering, University of Georgia, Athens, GA, USA.; 4 National Geographic Society, Washington, DC, USA.; 5 Ocean Conservancy, Washington, DC, USA.

## Abstract

Plastic waste affects environmental quality and ecosystem health. In 2010, an estimated 5 to 13 million metric tons (Mt) of plastic waste entered the ocean from both developing countries with insufficient solid waste infrastructure and high-income countries with very high waste generation. We demonstrate that, in 2016, the United States generated the largest amount of plastic waste of any country in the world (42.0 Mt). Between 0.14 and 0.41 Mt of this waste was illegally dumped in the United States, and 0.15 to 0.99 Mt was inadequately managed in countries that imported materials collected in the United States for recycling. Accounting for these contributions, the amount of plastic waste generated in the United States estimated to enter the coastal environment in 2016 was up to five times larger than that estimated for 2010, rendering the United States’ contribution among the highest in the world.

## Teaser

Plastic waste from the United States contaminates the environment domestically and in countries processing material for recycling.

## INTRODUCTION

Plastic waste contaminates all major ecosystems on the planet, with concern increasing about its potential impacts on wildlife and human health, as smaller and more widespread plastic particles are identified in both the natural (*1*–*4*) and built (*5*–*7*) environment. For decades, scientists have documented plastic debris in the ocean (*8*). Marine sources of ocean pollutants were addressed in the 1970s (*9*) and 1980s (*10*), before the focus turned to land as the purported, yet poorly substantiated, source of 80% of marine debris. In 2015, Jambeck *et al*. (*11*) used global solid waste management data compiled by the World Bank (*12*) to estimate the amount of inadequately managed plastic waste generated within 50 km of the coastline that entered the global ocean in 2010 [4.8 to 12.7 million metric tons (Mt)]. Since then, a nominal value of 8 Mt has been broadly adopted as a quantitative benchmark of the annual scale of ocean plastic pollution, spurring responses by nongovernmental organizations, policy-makers, and the plastics and consumer products industries. Stemming from this analysis, many remediation efforts have focused on countries in South and Southeast Asia (*13*–*15*).

However, high-income countries such as the United States and members of the European Union (EU-28) also had large plastic emissions to the ocean in 2010, according to Jambeck *et al*. (hereafter “2010 analysis”). Despite having robust waste management systems, the large coastal populations and very high per capita waste generation rates in these high-income countries together resulted in large amounts of mismanaged waste due only to litter (estimated 2% of waste generation) that is available to enter the ocean. According to the 2010 analysis, the U.S. coastal population generated the highest mass of plastic waste of any country (13.8 Mt, 112.9 million people), whereas coastal populations in EU-28 countries collectively produced even more plastic waste (14.8 Mt, 187.3 million people). The next highest country in coastal plastic waste generation was China (11.6 Mt per day, 262.9 million people).

Further, the United States is the second largest exporter of plastic scrap globally (*16*). If imported material is not properly managed in the receiving country, environmental inputs of plastic waste generated in the United States may be much higher than previously assessed. Here, we estimate plastic waste generation by the entire U.S. population in 2016, as well as the amount of plastic waste illegally dumped domestically and the amount likely to have been inadequately managed in countries that imported materials collected in the United States for recycling, both of which contribute plastic waste to land and the ocean.

## RESULTS AND DISCUSSION

From 2010 to 2016, global plastic production increased 26% from 334 to 422 Mt (*17*) and the proportion of plastics in solid waste grew from 10 to 12% globally, reaching 242 Mt in 2016 (*12*, *18*). Using updated waste generation and characterization data reported by the World Bank for 217 countries (*18*), and additional data available for the United States (see Materials and Methods), we calculated plastic waste generation in 2016 by total population of each country (Table 1). By both the World Bank estimate (34.0 Mt) and our refined U.S. estimate (42.0 Mt), in 2016, the U.S. population produced the largest mass of plastic waste of any country in the world and also had the largest annual per capita plastic waste generation of the top plastic waste–generating countries (>100 kg). The countries with the next highest plastic waste generation were also those with the highest populations, India and China, while EU-28 countries collectively generated more plastic waste than either India or China, despite having only ~40% of the population. Even in the EU-28, the per capita plastic waste generation rate was approximately half that of the United States.

**Table 1:** Countries with the highest plastic waste generation in 2016. Calculations using data reported in (*18*), with a refined estimate for the United States (bold text). EU-28 countries are reported collectively (italics).

**Country**	**Plastic waste** **generation** **(metric tons)**	**Total waste** **generation** **(metric tons)**	**% Plastic in** **solid waste**	**2016 Population** **(millions)**	**Per capita plastic** **waste generation** **(kg/year)**
**United States**	**42,027,215**	**320,818,436**	**13.1**	**323.1**	**130.09**
United States	34,020,748	263,726,732	12.9	323.1	105.30
*EU-28*	*29,890,143*	*243,737,466*	*11.7*	*511.2*	*54.56*
India	26,327,933	277,136,133	9.5	1,324.5	19.88
China	21,599,465	220,402,706	9.8	1,378.7	15.67
Brazil	10,675,989	79,081,401	13.5	206.2	51.78
Indonesia	9,128,000	65,200,000	14.0	261.6	34.90
Russian Federation	8,467,156	59,585,899	14.2	144.3	58.66
Germany	6,683,412	51,410,863	13.0	82.3	81.16
United Kingdom	6,471,650	32,037,871	20.2	65.6	98.66
Mexico	5,902,490	54,151,287	10.9	123.3	47.86
Japan	4,881,161	44,374,189	11.0	127.0	38.44
Thailand	4,796,494	27,268,302	17.6	69.0	69.54
Korea, Rep.	4,514,186	18,576,898	24.3	51.2	88.09
Italy	3,365,130	29,009,742	11.6	60.6	55.51
Egypt, Arab Rep.	3,037,675	23,366,729	13.0	94.4	32.16
France	2,929,042	32,544,914	9.0	66.9	43.81
Pakistan	2,731,768	30,352,981	9.0	203.6	13.42
Argentina	2,656,771	18,184,606	14.6	43.6	60.95
Algeria	2,092,007	12,378,740	16.9	40.6	51.59
Malaysia	2,058,501	13,723,342	15.0	30.7	67.09
Spain	1,832,533	20,361,483	9.0	46.5	39.42

To estimate the amount of plastic waste that could potentially enter the environment, we followed a similar process to the 2010 analysis, in which reported waste treatment and disposal methods (*18*) were used to calculate the proportion of plastic waste that is mismanaged, defined as not properly captured and contained (Table 2). For the United States, as reported by the U.S. Environmental Protection Agency (EPA) using a material flow methodology, plastic waste was treated by disposal in landfills (75.4%), incineration (15.3%), and recycling (9.3%) (*19*). By this estimation, 100% of waste generated in the United States was properly managed. While the material flow framework is useful to assess how managed waste is processed, it does not consider litter or illegal dumping or the fate of waste once it has been collected for recycling.

**Table 2:** Defined terms used in this study and estimated values for each treatment category for U.S. plastic waste generated in 2016. Mt, million metric tons.

**Terms used in** **this study**	**Definition**	**Treatment of U.S.** **plastic waste in** **2016 (Mt)**
Inadequately managed waste	Solid waste that is not collected and/or properly contained because of lack of waste management infrastructure	0
Litter	Solid waste that is intentionally or unintentionally disposed into the environment despite the availability of waste management infrastructure	0.84
Illegal dumping	Disposal of waste in an unpermitted area (*37*) despite the availability of waste management infrastructure	0.14–0.41
Exported, inadequately managed plastic waste	Plastic waste collected for recycling in the United States that was exported to countries where it was inadequately managed	0.15–0.99
Mismanaged waste	Sum of above categories	1.13–2.24

As in the 2010 analysis, we assumed a 2% litter rate because litter studies rarely report mass. We estimated the mass of plastic waste that is illegally dumped using available data from three locations (San Jose, CA; Sacramento, CA; and Columbus, OH) to compute an annualized per capita illegal dumping rate ranging from 0.43 to 1.28 kg, giving an estimated 0.14 to 0.41 Mt of illegally dumped plastic waste annually. Together with litter, the estimated amount of mismanaged plastic waste in the United States in 2016 was between 0.98 and 1.26 Mt, or 2.33 and 2.99% of plastic waste generated. We also calculated the proportion of mismanaged waste in the 50 countries with the highest plastic waste generation by total population in 2016 and assessed changes since 2010 (see the Supplementary Materials; table S6).

Solid waste assessments, such as that by the U.S. EPA (*19*), report the intended treatment of collected materials. Proper sanitary landfill and incineration treatments prevent solid waste from entering the environment (by-products including leachate, ash, and airborne emissions notwithstanding). However, reported recycling typically assesses the material collected for reprocessing (in the United States or elsewhere), rather than the final conversion into new materials. Consequently, the handling, transport, and processing after the initial collection allow for potential downstream leakage to the environment. In 2016, approximately 50% of plastic waste collected for recycling (hereafter “plastic scrap”) in countries around the world was traded internationally (*16*). In 2016, the United States exported 1.99 Mt of plastic scrap to 89 trade partners (*20*). More than 88% was exported to countries with greater than 20% inadequately managed waste, with the vast majority exported to China and Hong Kong (table S1). Contamination of materials collected for recycling is unavoidable because of the difficulty and associated high cost of sorting plastics by resin type, especially in “single stream” recycling programs where materials are mixed during collection and then mechanically and manually separated at a materials recovery facility (MRF) (*21*). Separated material compressed into bales is classified by grade, defined by contamination specifications, to standardize the commodity trading of these materials. For example, grade C specifications allow up to 27% contamination in bales of polyethylene terephthalate (PET) bottles and 20% for high-density polyethylene (HDPE) bottles, two higher-value plastics (*22*). Thus, baled plastic scrap must be further processed within an importing country to isolate the desired materials for recycling, while discarding the remainder as waste. Further, in 2016, the United States exported 19.75 Mt of paper and paperboard collected for recycling (hereafter “paper scrap”) to 77 trade partners (*20*). Contamination of paper scrap, including by thin plastic film and flattened plastic containers, reduces product quality and increases processing costs (*21*, *23*). Eighty-nine percent of paper scrap was exported to countries with greater than 20% inadequately managed waste (table S2).

To our knowledge, no quantitative estimates exist of the proportion of material exported for recycling that is ultimately discarded as waste or of the methods of disposal. From composition studies of plastic and paper scrap bales from MRFs in North America and the United Kingdom, we conservatively estimated that between 15 and 25% of material exported in plastic scrap bales, and 2 to 5% of material exported in paper scrap bales, consisted of low-value plastics and plastic waste that would likely have been discarded by processing facilities in importing countries. Investigative reports in Malaysia and Indonesia (*24*, *25*) describe massive amounts of processed waste disposed by open dumping and open burning. Without robust data to assess how widespread these practices are within and across the countries that import scrap materials from the United States, we applied a credible range estimate of between 25 and 75% of plastic waste discarded during the processing of plastic and paper scrap that was inadequately managed in receiving countries that have greater than 20% inadequately managed waste. This contributes an additional 0.15 to 0.99 Mt of plastic waste generated in the United States that likely entered the environment.

For comparison to the 2010 analysis and to inform potential leakage to the ocean, we calculated the amount of mismanaged waste generated by coastal populations in the 50 countries that generated the highest amounts of plastic waste in 2016. For the United States, we included upper- and lower-bound estimates of illegal dumping and inadequate management of exported scrap, as described above. We included exports in the coastal calculation because most of this material was transported to importing countries by ship where, upon arrival and to minimize additional transport cost, it was likely processed in close proximity to the coast. By our upper-bound estimate, in 2016, the United States was the third largest contributor of mismanaged plastic waste to the coastal environment globally (Table 3), representing an 82 to 400% increase from the 2010 estimate (0.28 Mt in 2010).

**Table 3:** Countries with the highest mismanaged plastic waste generated by coastal populations in 2016. The two U.S. estimates (bold text) provide lower and upper bounds reflecting contributions from domestic litter (0.31 Mt), domestic illegal dumping (0.05 to 0.15 Mt), and inadequate management of plastic waste generated during the processing of imported U.S. plastic and paper scrap in countries with greater than 20% inadequately managed waste (0.15 to 0.99 Mt). Mt, million metric tons. HIC, high income; UMC, upper middle income; LMC, lower middle income.

**Country**	**Mismanaged** **plastic waste** **(Mt)**	**Income status**	**Coastal population** **(millions)**	**Per capita plastic** **waste generation** **(kg/day)**	**% Plastic in** **solid waste**	**% Mismanaged** **waste**
Indonesia	4.28	LMC	202.49	0.68	14.0	61
India	3.16	LMC	201.20	0.57	9.5	79
**United States, upper bound**	**1.45**	**HIC**	**117.94**	**2.72**	**13.1**	**2.98**
Thailand	1.16	UMC	26.73	1.08	17.6	62
China	1.07	UMC	270.94	0.44	9.8	25
Brazil	1.03	UMC	78.68	1.05	13.5	25
Philippines	1.01	LMC	92.06	0.39	10.6	74
Egypt, Arab Rep.	0.71	LMC	24.82	0.68	13.0	90
Japan	0.67	HIC	114.26	0.96	11.0	15
Russian Federation	0.62	UMC	10.93	1.13	14.2	98
Vietnam	0.57	LMC	59.46	0.34	12.2	64
**United States, lower bound**	**0.51**	**HIC**	**117.94**	**2.72**	**13.1**	**2.33**
Bangladesh	0.36	LMC	75.87	0.28	4.7	97
Kuwait	0.35	HIC	3.03	1.59	20.0	100
Oman	0.35	HIC	3.83	1.18	21.0	100
Dominican Republic	0.33	UMC	8.83	1.11	10.0	94
Malaysia	0.33	UMC	24.90	1.23	15.0	20
Mexico	0.27	UMC	24.48	1.20	10.9	23
Argentina	0.26	HIC	17.58	1.14	14.6	25
Peru	0.25	UMC	14.67	0.77	10.5	58
Italy	0.25	HIC	34.59	1.31	11.6	13

Our study used global solid waste data reported by the World Bank that is more comprehensive than previously available. Compared to the preceding 2012 World Bank report (*12*), these data are more recent, are reported for more countries and economies, and include additional categories (e.g., for waste management practices). However, even with these marked improvements, in the absence of harmonized or standardized reporting, the variation in data collection and reporting methodologies may introduce errors or biases in the global comparative analysis (*18*). While such comparisons can be useful, it is most important to use robust, quantitative methods within a country to establish a baseline understanding of solid waste generation and management, against which future changes may be measured. In this study, we compiled data to refine U.S. estimates, yet variation between individual states in waste management systems and associated reporting may also introduce uncertainties in the nationwide analysis.

**Fig. 1:**
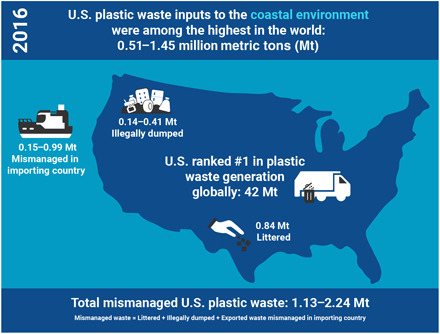
Schematic diagram illustrating the major quantitative results of the analysis of plastic waste generation in the United States in 2016 and the estimated mass of plastic waste that was mismanaged domestically by littering and illegal dumping and also abroad during processing of material collected for recycling in the United States that was exported to countries with greater than 20% inadequately managed waste (see the main text for details).

Our analysis demonstrates that the United States has contributed enormous amounts of plastic waste to the environment, including the ocean, despite having robust waste management infrastructure to collect, transport, and process waste (Fig. 1). The vast majority of U.S. residents have access to waste and recycling collection (*26*), yet illegal dumping and littering are still widespread, incurring high costs to municipalities for prevention and cleanup (*27*). Although the estimated amount of dumped material represents a small percentage of total waste generated, it is a large mass because the U.S. population generates the most solid waste of any country in the world. Although 9.3% of the plastic waste generated in the United States in 2016 was collected for recycling, the global market–driven export mainly to lower-income countries for processing ultimately resulted in substantial leakage of plastics to the environment. Since 2016, China has implemented a policy effectively prohibiting the import of “personal/household waste plastic,” “unsorted waste paper,” and other types of solid waste (*28*). Consequently, the total amounts of plastic and paper scrap exported from the United States in 2019 had fallen from 2016 values by 66 and 17%, respectively, with collective exports to China and Hong Kong dropping by 94% for plastic scrap and 60% for paper scrap. Further, in 2019, the Basel Convention was amended to include regulations on the global trade of plastic waste, specifically to address plastics entering the marine environment. The consequences of such policies on the management of this waste—whether domestically or when exported—and their related environmental impacts are unknown.

The United States has both the highest plastic waste generation rates in the world and a strong public desire to recycle these materials (*29*). The loss of export markets combined with reports of unacceptable handling of this waste abroad increasingly demonstrate the need for investment and overhaul of domestic infrastructure to manage this waste (*30*). Domestic recycling capacity has been increasing (*31*, *32*), and several pieces of federal legislation have been proposed to promote and enhance recycling programs (RECOVER Act of 2019 and RECYCLE Act of 2019), while others include policies such as minimum recycled content standards, a beverage container deposit scheme, and extended producer responsibility for packaging (Break Free from Plastic Pollution Act of 2020). Not only are the data in this study now several years old, but the economic, public health, and behavioral responses to the global COVID-19 pandemic have altered plastic waste generation, composition, and treatment practices in as-yet-unquantified ways in the United States and abroad, highlighting the urgency with which the United States must ensure proper treatment of its waste.

The most straightforward way to reduce environmental inputs of plastic waste is to produce less, especially waste that is not practicably or economically recyclable, readily escapes to the environment, or is unnecessary. Waste reduction must begin with material, product, and packaging design (*33*) that addresses end-of-life management, including an explicit cost for recovery and treatment. Ultimately, reducing plastic waste in the United States and assuming full responsibility for its reprocessing or disposal will require substantially greater commitments by resin producers, consumer products and retail companies, and the U.S. federal government.

## MATERIALS AND METHODS

### Estimating waste generation rates and percentage of plastic in municipal solid waste

The most recent World Bank report on the state of solid waste management globally (*18*) compiled data on municipal solid waste (MSW) for 217 countries around the world. They estimated MSW generation (metric tons per year) for 215 countries from reported data spanning multiple years (up to decades) and adjusted these estimates to the year 2016. For all countries except the United States, we used the 2016 adjusted MSW generation values reported in appendix A in (*18*). For all countries, we used the 2016 population data reported by the World Bank (https://data.worldbank.org/).

The 2016 adjusted MSW generation value reported by the World Bank for the United States is 263,726,732 metric tons (original source: U.S. EPA, 2014), which is equivalent to 2.24 kg per person per day. The U.S. EPA uses a materials flow methodology on the basis of production and expected product life span to estimate MSW generation. More direct estimates use waste disposal data from approximately 9000 U.S. MSW management facilities assembled and analyzed by the Environmental Research and Educational Foundation [EREF; (*34*)], which estimated that 342.6 million tons (310.8 Mt) of MSW was managed in 2010 and 347.0 million tons (314.8 Mt) was managed in 2013. The 2013 estimate is 37% higher than the 2013 estimate reported by the U.S. EPA and is equivalent to 2.72 kg per person per day. Further, a study based on waste quantity data from landfills and solid waste samples from 222 sites across the United States also found that the total mass of disposed MSW was significantly greater than the U.S. EPA estimate, consistent with the EREF result (*35*). Therefore, we used the EREF estimate of 2.72 kg person day for the U.S. MSW generation rate in 2016.

Country-level waste composition data were reported by the World Bank for nine material categories, including plastic (*36*). Plastic proportion was reported for 175 countries. For the 42 countries without data, percent plastic was estimated on the basis of average values reported according to income group (classified according to the World Bank estimates of 2015 gross national income per capita) and geographic region (classified by the World Bank) (table S3) (*18*).

The World Bank reported 12.9% plastic in U.S. waste using 2014 estimates reported by the U.S. EPA. In a more recent report, the U.S. EPA determined that, in 2016, plastics represented 13.1% of total MSW generation (*19*); this is the proportion we used in our analysis.

### Estimating proportion of inadequately managed waste according to country

The World Bank reported waste treatment data as percentages in 11 categories (*18*). Waste reported in “anaerobic digestion,” “compost,” “controlled landfill,” “sanitary landfill,” “recycling,” and “incineration” treatment categories was considered to be properly managed—that is, captured and contained to prevent leakage into the environment. Waste reported in “open dump,” “waterways,” “unaccounted for,” and “other” categories was considered to be inadequately managed. As reported in (*18*), waste not accounted for by any disposal method was considered dumped, and “other” usually referred to open burning of waste. The final category, “landfill unspecified,” is ambiguous, yet 63 countries spanning all income levels had reported values in this category ranging from 0.21 to 100% of waste treatment. With no further information available, we assumed that the proportion of waste in the landfill unspecified category that was inadequately managed is a function of income level, with 0, 25, 50, and 75% assumed to be inadequately managed in high-, upper-middle–, lower-middle–, and lower-income countries, respectively. Waste treatment data were not reported for 43 countries, of which only Venezuela had plastic waste generation greater than 1 Mt in 2016. Therefore, we did not estimate inadequately managed waste for countries with missing data, and they were omitted from the remainder of the analysis.

We attempted to refine the estimate of litter, which was crudely estimated as 2% of MSW generation in the 2010 analysis (*11*). We identified more recent litter studies in the United States and elsewhere; however, the lack of standard—or even comparable—methodologies across studies was prohibitive. In almost all studies, data were reported as piece counts, not as mass, and the size of litter counted was variable. Further, most studies in the United States quantify litter on a 15-foot-wide strip adjacent to roadways, which does not capture litter distributed across the wider landscape. For these reasons, we continued to assume that litter is 2% of MSW generation in all countries considered in the study.

### Estimating illegal dumping in the United States

Illegal dumping, defined as disposal of waste in an “unpermitted area,” is a recognized problem throughout the United States (*37*). Common materials found at illegal dumpsites include household trash, furniture, appliances, yard waste, construction and demolition waste, and automobile parts, including tires. We reviewed available illegal dumping studies in the United States for quantitative information about the mass and composition of illegally dumped waste. The best available data were from an illegal dumping survey in 40 census tracts spanning four income categories (derived from median household income data) in San Jose, CA, in 2016 (*38*). For each income category, debris mass was reported according to debris type (21 categories). We applied estimates of percent plastic composition (lower and upper bounds) for each category (table S4) and calculated the mass of illegally dumped plastic debris per person using the population size for each census tract. We computed a per capita dumping estimate for each income category and then averaged across all income categories, giving 0.108 to 0.321 kg of plastic per person. Because this was a one-time survey, we could not calculate an accumulation rate directly from reported data.

Data from two other reports allowed us to calculate additional estimates of the per capita mass of illegally dumped waste per year. Data compiled for Sacramento, CA, included the number of reported illegal dumping incidents per year for every 100 people and the average amount of waste collected per incident, for both Sacramento City and County in 2017 (*27*). Using these figures and our estimated mean plastic proportion of illegally dumped waste (22%), we calculated an illegal dumping rate of 0.50 kg of plastic per person per year in Sacramento County and 1.85 kg of plastic per person per year in Sacramento City.

Further, the city of Columbus, OH, began surveying and tracking incidences of illegally dumped waste in January 2019. From January 7 through April 12 (96 days), the city reported collecting an average of 15.7 tons per day (table S5). Using the U.S. Census population estimate for Columbus, OH (892,533 people on 1 July 2019) and our estimated median plastic proportion of illegally dumped waste (22%), we calculated an estimated illegal dumping rate of 1.28 kg of plastic per person per year.

The Sacramento and Columbus estimates of illegal dumping per capita per year are remarkably consistent. If the San Jose estimate represents accumulation over 3 months, then the per capita dumping rate is 0.43 to 1.28 kg of plastic per person per year, also consistent with these estimates. The assumption that dumpsites in San Jose are cleaned up within a 3-month period is reasonable because it was reported that GreenTeam of San Jose had a dedicated time each week (Saturdays between 6 a.m. and 3 p.m.) to clean up illegally dumped debris (*38*). The range in per capita plastic dumping rate (0.43 to 1.28 kg per person per year) was scaled by the size of the U.S. population to estimate the mass of illegally dumped plastic waste in 2016, giving 139,900 to 414,600 metric tons, which is equivalent to 0.33 to 0.99% of plastic waste generation.

### Estimating the quantity of material exported from the United States that is likely to have been discarded as plastic waste in importing countries that have >20% inadequately managed waste

We downloaded trade data from the United Nations Comtrade Database for the year 2016 (*20*). We selected U.S.-reported exports of “waste, parings and scrap, of plastic” (code 3915; hereafter “plastic scrap”) to all trade partners (89 total). For seven trade partners with no reported value for net weight of U.S. plastic scrap exports (China, China–Hong Kong SAR, Philippines, United Kingdom, Singapore, Ireland, and France), we downloaded the 2016 imports of plastic scrap from the United States reported by these countries. In total, we calculated that, in 2016, the United States exported 1.99 Mt of plastic scrap globally.

We calculated the mass of plastic scrap exported to countries with greater than 20% inadequately managed waste (assessed by methods described above). We assumed that Hong Kong had the same proportion of inadequately managed waste as China (23.25%) because, from Hong Kong’s reported trade data, Hong Kong’s re-export of plastic scrap to China accounts for 93.4% of the total mass of plastic scrap Hong Kong imported from all countries in 2016. We also assumed that “Other Asia nes” had 23.25% inadequately managed waste. The total mass of plastic scrap exported by the United States to countries with greater than 20% inadequately managed waste was 1.76 Mt, which is 88.7% of the total plastic scrap exported by the United States in 2016. Together, exports to Hong Kong and China accounted for 83.8% of the plastic scrap exported to countries with greater than 20% inadequately managed waste in 2016. Hereafter, we only consider the plastic scrap that was exported from the United States to countries with greater than 20% inadequately managed waste.

#### Estimating the proportion of plastic scrap that is likely to have been discarded by processing facilities in importing countries

We used data from a study by the Association of Postconsumer Plastic Recyclers (APR) conducted to determine the composition of material in mixed rigid plastic bales available for recycling in North America (*39*). In this study, 23 bales of post-consumer rigid plastic processed at four facilities in North America were analyzed according to 12 product categories (e.g., bottles, containers, bulky items, etc.) and 11 resin types. We estimated the proportion of bales composed of resins we deemed to be of relatively high value for recycling [HDPE, HDPE compatible/other, LDPE (low-density polyethylene), PP (polypropylene), PP compatible/other, mix PE/PP, and PET] and those deemed to be of relatively low value [PS (polystyrene), PVC (polyvinyl chloride), PC (polycarbonate), PLA (polylactic acid)/bio, other, plastic with metal, small plastic pieces, and trash]. We assumed that plastics comprised 50% of the material in the “trash” category and that low-value plastics and plastic trash would be discarded by a facility processing bales for recycling. Hereafter, “discarded plastics” refers to the sum of the proportions of low-value plastics and plastic trash.

Depending on the bale type (10 categories including All Rigid Plastic with and without Bulky, Pre-picked Rigid Plastic with and without Bulky, Tubs and Lids, Bulky Rigid Plastic, HDPE Injection-Bulky, HDPE Bottles and Containers, and PP Bottles and Containers), the proportion of discarded plastics ranged from 2.3% (in HDPE Bottles and Containers) to 52.6% (in one of two Pre-picked Rigid Plastic with Bulky categories). The average proportion of discarded plastics across all categories was 18.5%, and the weighted average according to number of bales analyzed was 17.0%.

Further, in a report on post-consumer non-bottle rigid plastic recycling in 2014, the largest export category (of six categories) was Pre-Picked Rigid Plastic (*40*). The average proportion of discarded plastics across the three Pre-Picked Rigid Plastic categories in the APR study ranged from 21.3 to 52.6%. On the basis of these data, we conservatively assumed that between 15 and 25% of plastic scrap exported from the United States consisted of low-value plastics and plastic trash that would likely have been discarded by processing facilities in importing countries.

#### Estimating the quantity of plastic in paper scrap exported from the United States that is likely to have been discarded in importing countries that have >20% inadequately managed waste

We downloaded trade data from the United Nations Comtrade Database for the year 2016 (*20*). We selected U.S.-reported exports of “waste and scrap of paper and paperboard” (code 4707; hereafter “paper scrap”) to all trade partners (77 total). In total, we calculated that, in 2016, the United States exported 19.75 Mt of paper scrap globally.

We calculated the mass of paper scrap exported to countries with greater than 20% inadequately managed waste (assessed by methods described above). We assumed that Hong Kong and “Other Asia nes” had the same proportion of inadequately managed waste as China (23.25%). The total mass of paper scrap exported by the United States to countries with greater than 20% inadequately managed waste was 17.54 Mt, which is 88.8% of the total paper scrap exported by the United States in 2016. Hereafter, we only consider the paper scrap that was exported from the United States to countries with greater than 20% inadequately managed waste.

We used data from a study conducted to assess the composition of post-consumer material sent to MRFs and the composition of material processed by the MRFs and sent to reprocessors (*41*). In the study, 85.7 metric tons of material sent to 18 MRFs in the United Kingdom, and 179.8 metric tons of material output from these MRFs, was hand-sorted and characterized according to 18 material categories. We estimated the proportion of plastics (in nine material categories, including two “Misc” categories that were assumed to contain 50% plastics) in the output streams of mixed paper, cardboard, and newspaper and periodicals and magazines (PAMs). Plastic contamination was estimated to be 5.0% in mixed paper, 2.1% in cardboard, and 1.3% in newspaper and PAMs in material output by MRFs for sale to reprocessors. On the basis of these data, we assumed that between 2 and 5% of paper scrap exported from the United States was contaminated with plastics that would likely have been discarded by processing facilities in importing countries.

#### Estimating the quantity of discarded plastic from imported plastic scrap and paper scrap that was inadequately managed in importing countries

The authors of this study (T.R.S. and J.R.J.) and investigative journalists (*24*, *25*) have visited plastic scrap processing facilities in a number of countries in this study (e.g., Vietnam, Indonesia, Malaysia, Mexico, Jordan, South Africa, and Belize) and have observed and documented open dumping and open burning of discarded plastics. However, there are no robust data to indicate how widespread these practices are within and across the countries that import scrap materials from the United States considered in this study. For this reason, we applied a credible range estimate of between 25 and 75% of discarded plastic from plastic scrap and paper scrap imported from the United States that was inadequately managed in the receiving country.

## Supplementary Material

abd0288_SM.pdf

abd0288_Data_S1.xlsx

abd0288_Table_S6.docx

## SUPPLEMENTARY MATERIALS

Supplementary material for this article is available at http://advances.sciencemag.org/cgi/content/full/6/44/eabd0288/DC1
