# Supplementary material for: The United States’ contribution of plastic waste to land and ocean
Source: Sci Adv. 2020 Oct 30;6(44):eabd0288. doi: 10.1126/sciadv.abd0288 (PMC7608798; doi:10.1126/sciadv.abd0288)
Supplement: abd0288_SM.pdf [file abd0288_SM.pdf]

Science Advances
AAAS

advances.sciencemag.org/cgi/content/full/6/44/eabd0288/DC1

Supplementary Materials for

The United States' contribution of plastic waste to land and ocean

Kara Lavender Law*, Natalie Starr, Theodore R. Siegler, Jenna R. Jambeck, Nicholas J. Mallos, George H. Leonard

*Corresponding author. Email: klavender@sea.edu

Published 30 October 2020, *Sci. Adv.* **6**, eabd0288 (2020)
DOI: 10.1126/sciadv.abd0288

The PDF file includes:

Supplementary Text
Tables S1 to S5
Legend for table S6
Legend for data S1
References

Other Supplementary Material for this manuscript includes the following:

(available at advances.sciencemag.org/cgi/content/full/6/44/eabd0288/DC1)

Table S6
Data S1

Supplementary Text

We calculated the proportion of mismanaged waste in the 50 countries with the highest plastic waste generation by total population in 2016 (see Materials and Methods section) and assessed changes in this proportion since 2010 (as reported in (*11*)). China, Indonesia, Vietnam, Malaysia, South Africa and Algeria all reported substantial decreases (22−58%) in mismanaged waste, while the largest increases (21−98%) were in Russia, Dominican Republic, Haiti, Guatemala, Peru, Turkey and multiple countries in the Middle East and North Africa (MEA) region, including Kuwait, Oman, United Arab Emirates, Syria, Iraq, Iran and Egypt. Because few countries reported disposal methods in 2010 (*12*), the 2010 analysis used a logistic regression model to estimate the proportion of inadequately managed waste based upon geographic region and economic status. Countries in the MEA region deviated from the predicted inverse relationship between income and proportion of inadequately managed waste; thus, it is not surprising that many of the countries in which substantial changes were reported since 2010 are in this region.

The top five generators of mismanaged plastic waste in the 2010 analysis (ranked by mass generated by coastal population) – China, Indonesia, Philippines, Vietnam and Thailand – all reported improved waste management in 2016, by 51%, 22%, 9%, 24% and 13%, respectively. Six of 14 high-income EU-28 countries with 0% inadequately managed waste (not including litter) in the 2010 analysis reported small contributions (1-3%) in the 2016 categories "Other" and "Unaccounted for", while Italy reported 11%. The U.S. reported 0% inadequately managed waste in 2010 and 2016.

According to 2016 estimates for total country populations, India was the largest generator of mismanaged plastic waste (20.8 Mt), followed by Russia, Indonesia, China and Thailand (Table S5). Since 2010, India's plastic waste generation increased due to an increased per capita waste generation rate (+69%) and an increase in the proportion of plastic in the waste stream (+6.5%, based upon income level and geographic region averages because data were not reported), both of which may be associated with industrialization, urbanization and economic growth (*42*), while India's proportion of mismanaged waste remained high (79%). China, on the other hand, reported a 60% decrease in waste generation rates and a 51% decrease in inadequate management from an estimated 74% in 2010 to 23% in 2016, with only 8% of waste reported in open dumps. China's decreased waste generation rate may be due to a change in estimation method from one using urban populations (*43*) to one incorporating lower rural waste generation rates. The decrease in inadequately managed waste can be explained by rapid development of incineration plants to manage municipal solid waste (*44*, *45*) and possibly also an expansion of controlled landfills (World Bank reported 60% treatment in "unspecified" landfills, which could neither be classified as open dump nor controlled landfill; *18*). In southeast Asia, both Philippines and Vietnam reported decreased per capita waste generation rates, reduced percent plastic in the waste stream, and improved waste management since 2010, resulting in a mass of

mismanaged plastic waste generated by the total population of each country in 2016 that is less than that generated only by their coastal populations in 2010.

It must be noted that the informal sector is active in collection and recycling of waste, especially in urban populations in low- and middle-income countries, with more than 15 million people globally working in this sector (*18*). These activities could not be systematically accounted for in this analysis, but may affect proportions of mismanaged waste reported here. Finally, as demonstrated by the examples above, the accurate estimation of mass of mismanaged plastic waste relies on all four factors: population size, per capita waste generation rate, proportion of plastic in the waste stream, and waste management practices, as well as robust data collection and reporting.

Table S1. United States trade partners for "Waste, parings and scrap, of plastics" (Code 3915 in Comtrade database) in 2016 that had greater than 20% inadequately managed waste, and the mass of plastic scrap imported by these countries from the U.S. in tonnes (t).

2016 Trade partner	Mass of imported plastic scrap (t)	% Inadequately managed waste
China, Hong Kong SAR	783,938	23
China	693,447	23
India	91,913	77
Vietnam	66,747	62
Indonesia	34,521	59
Mexico	32,830	21
Other Asia, nes	30,521	23
Turkey	6,606	45
Thailand	6,541	60
El Salvador	5,341	22
Guatemala	2,859	75
Dominican Republic	1,762	92
Pakistan	1,640	70
Bangladesh	1,370	95
Philippines	872	72
Nigeria	797	80
Honduras	705	29
Peru	346	56
Ecuador	231	34
Nicaragua	88	90
Cayman Islands	70	79
Brazil	68	23
United Arab Emirates	49	62
Myanmar	41	100
Bolivia	37	56
Romania	34	31
Guyana	32	53
Samoa	28	33
Panama	24	42
Argentina	23	23
Jamaica	21	36
Sri Lanka	20	85
Morocco	19	54
Nepal	18	88
Kuwait	11	100
Jordan	9	57
Mauritania	9	55
Yemen	7	80
Suriname	3	100
Lebanon	3	29

Trinidad and Tobago	3	87
Aruba	1	89
China, Macao SAR	1	80
Belize	1	66
Haiti	< 1	90

Table S2. United States trade partners for "Waste and scrap of paper and paperboard" (Code 4707 in Comtrade database) in 2016 that had greater than 20% inadequately managed waste, and the mass of paper scrap imported by these countries from the U.S. in tonnes (t).

2016 Trade Partner	Mass of imported paper scrap (t)	% Inadequately managed waste
China	13,189,751	23
India	1,561,924	77
Mexico	1,496,470	21
Indonesia	379,594	59
Thailand	293,769	60
Other Asia, nes	255,590	23
Vietnam	120,673	62
El Salvador	40,270	22
Ecuador	29,429	34
Pakistan	27,160	70
Philippines	25,257	72
Guatemala	22,397	75
Peru	17,954	56
Argentina	16,341	23
Turkey	11,654	45
Trinidad and Tobago	8,455	87
China, Hong Kong SAR	8,369	23
Dominican Republic	8,275	92
Bolivia	7,313	56
Panama	4,696	42
Honduras	3,276	29
Brazil	2,968	23
Egypt	1,901	88
Lebanon	1,469	29
Bangladesh	940	95
Suriname	754	100
United Arab Emirates	745	62
Nicaragua	468	90
Tunisia	453	56
United Rep. of Tanzania	310	100
Morocco	268	54
Paraguay	266	23
Belize	212	66
Botswana	130	99
Iraq	124	100
Kenya	119	92
Jordan	52	57
Congo	34	74
Montenegro	21	95

Table S3. Percent plastic in municipal solid waste computed from average of values reported according to income level and region (*18*).

	**Income level**			
	High	Upper-middle	Lower-middle	Low
East Asia and Pacific	12.50	11.50	11.50	9.20
Europe and Central Asia	12.25	11.25	11.25	8.95
Latin America and the Caribbean	12.50	11.50	11.50	9.20
Middle East and North Africa	12.50	11.50	11.50	9.20
North America	12.50	11.50	11.50	9.20
South Asia	10.50	9.50	9.50	7.20
Sub-Saharan Africa	10.80	9.80	9.80	7.50

Table S4. Categories of debris type in San Jose illegal dumping study (*38*) with estimated ranges of the minimum and maximum proportion of plastic we assumed for each category.

Waste category	Minimum % plastic	Maximum % plastic
Mixed rigid plastics	100	100
Polystyrene	100	100
Child/baby/elderly items	13.1	95
Mattress*	20	75
Mixed textiles	10	75
Patio/garden/pool	25	75
Furniture	10	50
RV/mobile home	20	50
Mixed recyclables	25.5	25.5
Appliances†	10	25
Exercise equipment	10	25
Tire‡	11	24
Car/auto parts	5	20
Electronics§	17	17
Garbage bag	13.1	13.1
Homeless encampment	13.1	13.1
Construction & demolition‖	2	7
Yard trimmings	0	0
Scrap metal	0	0
Wood scraps	0	0
Hazardous waste	0	0

*https://www.rubbernews.com/article/20180516/NEWS/180519956/bed-in-a-box-mattress-market-expanding

†https://www.steel.org/steel-markets/appliances

‡https://www.ustires.org/whats-tire-0

§https://plastics.americanchemistry.com/Ten-Facts-About-Plastics-from-Electronics/

‖U.S. EPA, 1998. Characterization of Building-Related Construction and Demolition Debris in the United States.

Table S5. Number of Solid Waste Incidents (SWIs) and mass of illegally dumped waste (in short tons) collected by refuse crews from three facilities in Columbus, OH between January 7 and April 12, 2019. Data kindly provided by Columbus Refuse Division administrator, Timothy J. Swauger.

Refuse collection facility	# of SWIs	Waste collected [tons]
Stephen R. Lennon Center	146	501.82
Alum Creek	124	366.04
Georgesville Road	120	636.85

Table S6. Countries with the highest mismanaged plastic waste (in Mt) in 2016 calculated for total country population (gray shading). Calculations used data reported by World Bank (*18*) for all countries but the United States (bold text), for which a refined estimate for waste generation and percent plastic was used. The United States mismanaged proportion includes upper and lower bound estimates for illegal dumping. Unshaded columns indicate results of 2010 analysis (*11*). HIC = high income, UMC = upper middle income, LMC = lower middle income, LIC = low income, NR = not reported.

Table is in a separate Word file.

Data S1. Database of values used in the calculation of the mass of plastic waste generation and mismanaged plastic waste. Data for 217 countries as reported by World Bank (*18*), with additional estimates for the United States utilizing refined values for solid waste generation rate and percent plastic in solid waste. United States estimates of mismanaged plastic waste include contributions from domestic illegal dumping and inadequate management of plastic waste generated during processing of imported scrap materials from the U.S. in countries with greater than 20% inadequately managed waste (see text for details).

Data are in a separate Excel file.

REFERENCES AND NOTES

1. K. L. Law, Plastics in the marine environment. *Ann. Rev. Mar. Sci.* **9**, 205–229 (2017).

2. M. Wagner, S. Lambert, *Freshwater Microplastics* (Springer International Publishing, 2018).

3. Y. Chae, Y.-J. An, Current research trends on plastic pollution and ecological impacts on the soil ecosystem: A review. *Environ. Pollut*. **240**, 387–395 (2018).

4. M. Bergmann, S. Mützel, S. Primpke, M. B. Tekman, J. Trachsel, G. Gerdts, White and wonderful? Microplastics prevail in snow from the Alps to the Arctic. *Sci. Adv*. **5**, eaax1157 (2019).

5. R. Dris, J. Gasperi, V. Rocher, M. Saad, N. Renault, B. Tassin, Microplastic contamination in an urban area: A case study in Greater Paris. *Environ. Chem.* **12**, 592–599 (2015).

6. S. A. Carr, J. Liu, A. G. Tesoro, Transport and fate of microplastic particles in wastewater treatment plants. *Water Res*. **91**, 174–182 (2016).

7. Microplastics in drinking-water (World Health Organization, 2019).

8. P. G. Ryan, A brief history of marine litter research, in *Marine Anthropogenic Litter*, M. Bergmann, L. Gutow, M. Klages, Eds. (Springer Open, 2015), pp. 1–25.

9. ETATS-UNIS. National research council, National Research Council (U.S.). Study Panel on Assessing Potential Ocean Pollutants, National Research Council (U.S.). Ocean Affairs Board, *Assessing Potential Ocean Pollutants: A Report of the Study Panel on Assessing Potential Ocean Pollutants to the Ocean Affairs Board, Commission on Natural Resources, National Research Council* (National Academies, 1975).

10. International Convention for the Prevention of Pollution from Ships (MARPOL), Annex V: Prevention of Pollution by Garbage from Ships (International Maritime Organization, 1988).

11. J. R. Jambeck, R. Geyer, C. Wilcox, T. R. Siegler, M. Perryman, A. Andrady, R. Narayan, K. L. Law, Plastic waste inputs from land into the ocean. *Science* **347**, 768–771 (2015).

12. D. Hoornweg, P. Bhada-Tata, *What a Waste: A Global Review of Solid Waste Management* (World Bank, 2012).

13. *Stemming the Tide: Land-based strategies for a plastic-free ocean* (McKinsey & Company and Ocean Conservancy, 2015).

14. Alliance to End Plastic Waste; https://endplasticwaste.org.

15. Bangkok Declaration on Combating Marine Debris in ASEAN Region (ASEAN, 2019); https://asean.org/storage/2019/06/2.-Bangkok-Declaration-on-Combating-Marine-Debris-in-ASEAN-Region-FINAL.pdf.

16. A. L. Brooks, S. Wang, J. R. Jambeck, The Chinese import ban and its impact on global plastic waste trade. *Sci. Adv.* **4**, eaat0131 (2018).

17. R. Geyer, J. R. Jambeck, K. L. Law, Production, use, and fate of all plastics ever made. *Sci. Adv.* **3**, e1700782 (2017).

18. S. Kaza, L. Yao, P. Bhada-Tata, F. Van Woerden, *What a Waste 2.0: A Global Snapshot of Solid Waste Management to 2050* (World Bank Group, 2018).

19. U.S. Environmental Protection Agency (EPA), *Advancing Sustainable Materials Management: 2016 and 2017 Tables and Figures* (EPA, 2019).

20. United Nations Comtrade Database; https://comtrade.un.org/data.

21. H. Damgacioglu, M. Hornilla, O. Bafail, N. Celik, Recovering value from single stream material recovery facilities—An outbound contamination analysis in Florida. *Waste Manag.* **102**, 804–814 (2020).

22. *ISRI Scrap Specifications Circular* (Institute of Scrap Recycling Industries Inc., 2018).

23. R. Miranda, M. C. Monte, A. Blanco, Analysis of the quality of the recovered paper from commingled collection systems. *Resour. Conserv. Recycl.* **72**, 60–66 (2013).

24. The Recycling Myth: Malaysia and the Broken Global Recycling System (Greenpeace Malaysia, 2018).

25. S. Wilson, Here is away, *Resource Recycling News* (2019); https://resource-recycling.com/recycling/2019/09/09/here-is-away/.

26. 2015–16 Centralized Study on Availability of Recycling (Moore Recycling Associates Inc., 2016).

27. Report for Sacramento Regional Solid Waste Authority (Eunomia Research & Consulting, 2018).

28. Notice of the General Office of the State Council on Issuance of Reform and Implementation Plan to Enhance Solid Waste Import Management System by Prohibiting the Entry of Foreign Waste (Chinese Government Network, 2017); www.isri.org/docs/default-source/int'l-trade/170727chinaannouncement-en.pdf.

29. 2016 Pew Research Center's American Trends Panel: Survey questionnaire and topline (Pew Research Center, 2016).

30. M. J. Rogoff, D. E. Ross, The future of recycling in the United States. *Waste Manag. Res*. **34**, 181–183 (2016).

31. C. Staub, Domestic plastics recycling capacity is expanding, *Resource Recycling News* (2020); https://resource-recycling.com/recycling/2020/02/25/domestic-plastics-recycling-capacity-is-expanding/.

32. C. Staub, These projects are boosting domestic capacity for recycled paper, *Resource Recycling News* (2019); https://resource-recycling.com/recycling/2019/08/27/these-projects-are-boosting-domestic-capacity-for-recycled-paper/.

33. Twenty Questions About Design Behavior for Sustainability (International Expert Panel on Behavioral Science for Design, 2019); www.nature.com/documents/design_behavior_for_sustainability.pdf.

34. Municipal Solid Waste Management in the U.S.: 2010 & 2013 (Environmental Research & Education Foundation, 2016).

35. J. T. Powell, M. R. Chertow, Quantity, components, and value of waste materials landfilled in the United States. *J. Ind. Ecol.* **23**, 466–479 (2019).

36. What a Waste Global Database; https://datacatalog.worldbank.org/dataset/what-waste-global-database.

37. U.S. Environmental Protection Agency (EPA), *Illegal Dumping Prevention Guidebook* (EPA, 1998).

38. A. A. Brandt, Illegal Dumping as an Indicator for Community Social Disorganization and Crime, thesis, San Jose State University (2017).

39. National Mixed Rigid Plastic Bale Composition Study (The Association of Postconsumer Plastic Recyclers, 2015).

40. 2014 National Postconsumer Non-Bottle Rigid Plastic Recycling Report (Moore Recycling Associates Inc., 2016).

41. Enviros Consulting, *MRF Quality Assessment Study: Material quality assessment of municipal MRFs within the UK* (WRAP, 2009).

42. K. D. Sharma, S. Jain, Overview of municipal solid waste generation, composition, and management in India. *J. Environ. Eng.* **145**, 04018143 (2019).

43. D. Hoornweg, P. Lam, M. Chaudhry, *Waste Management in China: Issues and Recommendations* (World Bank Group, 2005).

44. L. Ji, S. Lu, J. Yang, C. Du, Z. Chen, A. Buekens, J. Yan, Municipal solid waste incineration in China and the issue of acidification: A review. *Waste Manag. Res*. **34**, 280–297 (2016).

45. J.-W. Lu, S. Zhang, J. Hai, M. Lei, Status and perspectives of municipal solid waste incineration in China: A comparison with developed regions. *Waste Manag*. **69**, 170–186 (2017).
