# Supplementary material for: The United States’ contribution of plastic waste to land and ocean
Source: Sci Adv. 2020 Oct 30;6(44):eabd0288. doi: 10.1126/sciadv.abd0288 (PMC7608798; doi:10.1126/sciadv.abd0288)
Supplement: abd0288_Table_S6.docx [file abd0288_Table_S6.docx]

**Table S6: Countries with the highest mismanaged plastic waste (in Mt) in 2016 calculated for total country population (gray shading).** Calculations used data reported by World Bank (*18*) for all countries but the United States (bold text), for which a refined estimate for waste generation and percent plastic was used. The United States mismanaged proportion includes upper and lower bound estimates for illegal dumping. Unshaded columns indicate results of 2010 analysis (*11*). HIC = high income, UMC = upper middle income, LMC = lower middle income, LIC = low income, NR = not reported.

| **Country** | **2016**  **Misman. plastic waste [Mt]** | **Income status** | **2016 Population**  **[millions]** | **2016**  **Waste generation [kg/per/day]** | **2010**  **Waste generation [kg/per/day]** | **2016 %Plastic** | **2010 %Plastic** | **2016 %Misman.** | **2010 %Misman.** | |
| --- | --- | --- | --- | --- | --- | --- | --- | --- | --- | --- |
| India | 20.80 | LMC | 1,324.5 | 0.57 | 0.34 | 9.5 | 3.0 | 79 | 87 |  |
| Russian Federation | 8.26 | UMC | 144.3 | 1.13 | 0.93 | 14.2 | 12.0 | 98 | 18 |  |
| Indonesia | 5.52 | LMC | 261.6 | 0.68 | 0.52 | 14.0 | 11.0 | 61 | 83 |  |
| China | 5.45 | UMC | 1,378.7 | 0.44 | 1.10 | 9.8 | 11.0 | 25 | 76 |  |
| Thailand | 2.99 | UMC | 69.0 | 1.08 | 1.20 | 17.6 | 12.0 | 62 | 75 |  |
| Egypt, Arab Rep. | 2.72 | LMC | 94.4 | 0.68 | 1.37 | 13.0 | 13.0 | 90 | 69 |  |
| Brazil | 2.69 | UMC | 206.2 | 1.05 | 1.03 | 13.5 | 16.0 | 25 | 11 |  |
| Pakistan | 1.97 | LMC | 203.6 | 0.41 | 0.79 | 9.0 | 13.0 | 72 | 88 |  |
| Nigeria | 1.36 | LMC | 186.0 | 0.51 | 0.79 | 4.8 | 13.0 | 82 | 83 |  |
| Mexico | 1.36 | UMC | 123.3 | 1.20 | 1.24 | 10.9 | 7.0 | 23 | 14 |  |
| Kazakhstan | 1.27 | UMC | 17.8 | 0.79 | NR | 25.0 | NR | 99 | NR |  |
| **United States** | **1.26** | **HIC** | **323.1** | **2.72** | **2.58** | **13.1** | **13.0** | **2.99** | **2** |  |
| Kenya | 1.20 | LMC | 49.1 | 0.38 | 0.30 | 18.7 | 9.0 | 94 | 85 |  |
| Philippines | 1.14 | LMC | 103.7 | 0.39 | 0.50 | 10.6 | 15.0 | 74 | 83 |  |
| Iran, Islamic Rep. | 1.11 | UMC | 79.6 | 0.61 | 1.20 | 8.5 | 12.0 | 74 | 52 |  |
| Congo, Dem. Rep. | 1.05 | LIC | 78.8 | 0.50 | 0.50 | 7.5 | 9.0 | 97 | 87 |  |
| **United States** | **0.98** | **HIC** | **323.1** | **2.72** | **2.58** | **13.1** | **13.0** | **2.33** | **2** |  |
| Vietnam | 0.90 | LMC | 93.6 | 0.34 | 0.79 | 12.2 | 13.0 | 64 | 88 |  |
| Myanmar | 0.86 | LMC | 53.0 | 0.38 | 0.44 | 11.5 | 17.0 | 100 | 89 |  |
| Tanzania | 0.81 | LIC | 53.1 | 0.56 | 0.26 | 7.5 | 9.0 | 100 | 86 |  |
| Japan | 0.75 | HIC | 127.0 | 0.96 | 1.71 | 11.0 | 10.0 | 15 | 2 |  |
